# A Protocol for Evaluating Digital Technology for Monitoring Sleep and Circadian Rhythms in Older People and People Living with Dementia in the Community

**DOI:** 10.3390/clockssleep6010010

**Published:** 2024-02-29

**Authors:** Ciro della Monica, Kiran K. G. Ravindran, Giuseppe Atzori, Damion J. Lambert, Thalia Rodriguez, Sara Mahvash-Mohammadi, Ullrich Bartsch, Anne C. Skeldon, Kevin Wells, Adam Hampshire, Ramin Nilforooshan, Hana Hassanin, The UK Dementia Research Institute Care Research & Technology Research Group, Victoria L. Revell, Derk-Jan Dijk

**Affiliations:** 1 Surrey Sleep Research Centre, University of Surrey, Guildford GU2 7XP, UK; k.guruswamyravindran@surrey.ac.uk (K.K.G.R.); g.atzori@surrey.ac.uk (G.A.); djlamber@ic.ac.uk (D.J.L.); u.bartsch@surrey.ac.uk (U.B.); v.revell@surrey.ac.uk (V.L.R.); d.j.dijk@surrey.ac.uk (D.-J.D.); 2 UK Dementia Research Institute Care Research & Technology Centre (CR&T), Imperial College London and the University of Surrey, London W12 0NN, UK; s.mahvash@qmul.ac.uk (S.M.-M.); a.skeldon@surrey.ac.uk (A.C.S.); k.wells@surrey.ac.uk (K.W.); ramin.nilforooshan@sabp.nhs.uk (R.N.);; 3 School of Mathematics & Physics, University of Surrey, Guildford GU2 7XH, UK; 4 Centre for Vision, Speech and Signal Processing, University of Surrey, Guildford GU2 7XH, UK; 5 Department of Brain Sciences, Imperial College, London W12 0NN, UK; a.hampshire@imperial.ac.uk; 6 Surrey and Borders Partnership NHS Foundation Trust Surrey, Chertsey KT16 9AU, UK; 7 Surrey Clinical Research Facility, University of Surrey, Guildford GU2 7XP, UK; 8 NIHR Royal Surrey CRF, Royal Surrey Foundation Trust, Guildford GU2 7XX, UK

**Keywords:** sleep, circadian, longitudinal, monitoring, ageing, evaluation, technology, dementia, good health, well-being

## Abstract

Sleep and circadian rhythm disturbance are predictors of poor physical and mental health, including dementia. Long-term digital technology-enabled monitoring of sleep and circadian rhythms in the community has great potential for early diagnosis, monitoring of disease progression, and assessing the effectiveness of interventions. Before novel digital technology-based monitoring can be implemented at scale, its performance and acceptability need to be evaluated and compared to gold-standard methodology in relevant populations. Here, we describe our protocol for the evaluation of novel sleep and circadian technology which we have applied in cognitively intact older adults and are currently using in people living with dementia (PLWD). In this protocol, we test a range of technologies simultaneously at home (7–14 days) and subsequently in a clinical research facility in which gold standard methodology for assessing sleep and circadian physiology is implemented. We emphasize the importance of assessing both nocturnal and diurnal sleep (naps), valid markers of circadian physiology, and that evaluation of technology is best achieved in protocols in which sleep is mildly disturbed and in populations that are relevant to the intended use-case. We provide details on the design, implementation, challenges, and advantages of this protocol, along with examples of datasets.

## 1. Introduction

### 1.1. The Need for Technology to Monitor Sleep and Circadian Rhythms Longitudinally

Sleep and the circadian system are important contributors to well-being and both physical and mental health [1,2,3]. Disruptions to sleep or circadian rhythms may be a predictor of and/or contributor to disease progression as well having a negative impact on quality of life. Much of our knowledge in this area is based on self-report, cross-sectional studies, or short-term laboratory studies. The capacity to unobtrusively monitor sleep-wake cycles and circadian rhythms over long periods of time at home offers the following opportunities: (a) early detection of decline and implementation of appropriate action, (b) monitoring disease progression and associated clinical outcomes, (c) increasing understanding of the relationship between sleep and circadian physiology and clinical symptoms, (d) monitoring the response to interventions. Longitudinal monitoring of sleep and circadian variables within an individual may also facilitate the development of personalised interventions.

People living with dementia (PLWD) and their caregivers are examples of populations that may benefit from monitoring of sleep and circadian rhythms over long periods of time. Disturbances of sleep and circadian rhythms are highly prevalent in dementia and include night-time awakening and wandering, long naps during the daytime, and early or late sleep timing [4,5,6,7]. Sleep timing disturbances may vary across dementias such as fronto-temporal dementia (FTD) and Alzheimer’s disease [8]. Sleep disorders are prevalent in dementia, particularly obstructive sleep apnoea and REM sleep behaviour disorder. These sleep disorders are risk factors for dementia and neurodegeneration and contribute to cognitive decline [9,10,11]. These disruptions not only affect the quality of life of PLWD but are also a burden to their care givers (e.g., [12,13,14,15]). Sleep and circadian disruption are a major contributing factor to PLWD being moved into care homes (e.g., [16,17,18]). These sleep and circadian disturbances may be a consequence of the neurodegenerative process and, as such, be an indicator of disease progression (e.g., [19]). Sleep disturbances may also drive disease progression and thus be a target for intervention. Some of the symptoms of dementia appear to be very sensitive to sleep disturbance. For example, the night-to-night variation in sleep continuity predicts the day-to-day variation in vigilance, cognition, memory, and behavioural problems in people with Alzheimer’s disease [20]. 

### 1.2. Gold-Standard Assessments of Sleep and Circadian Rhythms: Advantages and Disadvantages

Sleep: We can measure sleep in many different ways, from simple self-report (e.g., sleep diaries), to increasing complexity at the behavioural (e.g., bed occupancy) and physiological (e.g., EEG, cardiovascular) level. The classification of vigilance states can be made at the simple sleep vs. wake distinction, at the more detailed macrostructure of the different stages of non-rapid eye movement sleep (NREM) stages N1 to N3 and REM sleep, and finally at the microstructure of the electroencephalogram (EEG) signal, as reflected n power spectral density or other EEG measures. From these measurements a range of parameters to describe sleep can be derived: self-reported sleep quality, the timing of sleep within the 24-h day, total sleep time (TST), sleep onset latency (SOL), wake after sleep onset (WASO), sleep efficiency (SE), spectral power of different EEG frequency bands, and individual EEG events such as slow waves and sleep spindles as well as their phase relationships (e.g., [1,5]). 

The gold-standard method of assessing sleep is laboratory-based polysomnography (PSG) which is performed in accordance with guidelines of the American Academy of Sleep Medicine [21]. PSG is a comprehensive overnight physiological assessment including EEG, electrooculogram (EOG), electromyogram (EMG), electrocardiogram (ECG), oxygen saturation (SpO2), respiration effort and airflow, limb movement (EMG), body position, and video recording. The recordings can then be scored to provide a detailed picture of sleep structure and physiology, including the presence of any clinical sleep disorders such as sleep apnoea and periodic limb movements disorder. To try and reduce the burden to participants and the intensive staff requirement for the acquisition and analysis of PSG recordings, recently there has been a move to develop devices that utilise reduced montages as well as working on improving automated scoring algorithms.

Circadian rhythmicity: To understand the contribution of the circadian system to health and disease, it is necessary to be able to characterise its properties, which include the phase (timing) and amplitude (strength) of the rhythms. Traditionally, assessment of phase and amplitude has been achieved by acquiring time series data of gold standard measures (e.g., melatonin, cortisol, core body temperature) in highly controlled laboratory conditions (i.e., dim light, continual wakefulness, controlled posture, controlled calorie intake) [22].

The gold-standard sleep and circadian assessment approaches have a number of drawbacks: (a) high associated cost due to the requirement for participants to be supervised in a laboratory environment by appropriately skilled staff, (b) a high level of burden on participants due to amount of equipment that needs to be worn and needing to travel away from home to a laboratory setting, (c) they are potentially invasive if, for example, blood samples are collected, (d) they require technical analysis skills, e.g., for scoring the PSG recording, (e) they are unrepresentative of normal individual sleep patterns due to first night effects and novel controlled surroundings. Moreover, a single PSG recording and single melatonin profile in the laboratory only provides a snapshot of an individual’s sleep/circadian physiology and behaviour.

### 1.3. Technology for Monitoring Sleep and Circadian Rhythms at Home: Current and Novel Approaches

New digital health technology to monitor sleep/circadian behaviour and physiology at home is rapidly emerging on the consumer and research markets. These devices can potentially provide behavioural level data, including bed occupancy and activity, as well as sleep stages, heart rate, breathing rate, oxygen saturation, and may even quantify sleep apnoea. Some devices also measure environmental variables including light, noise, and air quality. Consumer monitoring devices are designed to appeal to the general public in terms of cost, appearance, and the information that they provide. However, as these are consumer rather than medical devices, no particular level of quantitative performance is mandated or guaranteed. Nonetheless, the ability to cost-effectively monitor sleep and circadian rhythms in an individual’s own home offers several advantages. In particular, the assessments are made in a natural environment and longitudinally which allow the influence of daily activities/behaviours and local environmental factors, including light and temperature, on sleep and circadian rhythms to be assessed. 

Longitudinal assessments of circadian rhythms and sleep, to date, have taken three approaches: (1) measuring rest-activity patterns with wrist-worn actigraphy in conjunction with sleep diary, and using sleep timing as a proxy for the phase of the circadian pacemaker, (2) assessment of circadian phase through sample collection and measurement of melatonin or its metabolites at defined intervals, (3) combining light and activity measurements with mathematical models to predict circadian phase and period and assess the relative contribution of environmental and biological factors to sleep phenotypes [22,23].

Actigraphy records limb movement activity (accelerometery) and then uses a proprietary algorithm to process this movement data to estimate whether an individual is awake or asleep for a defined epoch of time (e.g., 60 s), and subsequently derive sleep measures including TST, SOL and WASO. Importantly, the current guidelines recommend that for actigraphy to provide useful information, it should be combined with a daily sleep diary, which imposes a burden on the participants. In addition to assessment of information on sleep duration and efficiency, non-parametric analysis can be applied to determine a range of variables relevant to sleep regularity and circadian rhythmicity: inter-daily stability (IS), a measure of day-to-day consistency of activity patterns; intra-daily variability (IV), a measure of how much activity varies within a 24-h period; 10 h of highest activity (M10); 5 h of lowest activity (L5); and relative amplitude (M10: L5) [24]. However, the use of the timing of sleep/rest periods as an estimate for circadian phase is not advisable [2]. This is because the relationship between the circadian clock, as indexed by melatonin, and sleep timing varies in both healthy individuals [25] but also in different mental health conditions [2]. Furthermore, the phase relationship between sleep and circadian rhythms is relevant; for example, it predicts whether or not late sleep timing (eveningness) associates with depressive symptoms [3]. 

Field assessments of the gold-standard marker of circadian phase, i.e., melatonin profiles, are challenged by the fact that melatonin is sensitive to exogenous factors including environmental light and posture. Collection of saliva samples, under dim light whilst seated, at 30 min intervals in the 3–4 h before habitual bedtime allows the dim light melatonin onset (DLMO) to be determined as a marker of circadian phase. Implementation of technology, including containers that track when salivettes are removed for sampling, have allowed the development of home protocols that have been validated against DLMO collected in the laboratory [26]. An alternative approach that is less restrictive for participants is 48-h urinary collections to measure the urinary metabolite of melatonin, 6-sulphatoxy-melatonin (aMT6s). This methodology has been used successfully in both blind individuals and those living with schizophrenia, who frequently suffer circadian and sleep disruption, to track circadian phase over several weeks (e.g., [27,28]). It should be noted that this approach may cause burden to participants and, as the circadian parameters are computed from a rhythm derived from samples collected over 4–8-h bins, the markers may not have sufficient resolution to detect small but relevant changes in circadian phase. More recently, machine learning, statistical and mathematical models have been used to extract features from only a few samples of high dimensional data, e.g., transcriptomics, metabolomics, or longitudinal simultaneous recordings of light exposure, activity, and physiology [22]. For example, the interaction between the circadian system, sleep homeostat, and environmental light exposure approaches have been successfully applied to wearable data to predict circadian phase [29,30]. However, many of these approaches have yet to be tested and validated in different populations or under different sleep/wake, light/dark schedules.

### 1.4. Evaluating Technology: The Issues

The main issues with novel technology are the following: (1) lack of evaluation against gold standard measures, (2) if evaluation studies are performed then they are typically in young, healthy individuals for a habitual time-in-bed period where sleep efficiency is high and where rest/activity rhythms are robust and regular, (3) consumer device hardware and algorithms are constantly being updated which means that any evaluation that has been performed may be rapidly out of date.

The predominance of evaluation studies of young participants in laboratory studies means that the device performance may not translate to situations of disturbed sleep/circadian rhythms or in clinical/older populations who may benefit from long-term use of the devices. This is because sleep undergoes well-characterised changes with age, but also in dementia, at both the macro- and microstructure level. In addition to changes in sleep, ageing is associated with changes in the circadian system in terms of its timing, amplitude, and relationship with sleep (e.g., [31]), as well as changes in light exposure, crucial for stability and robustness of the circadian clock, due to changes in photic sensitivity (e.g., [32]) and the lived light environment (e.g., [33]). As such, circadian technologies may not perform as well in older individuals.

Nevertheless, there is a lack of evaluation studies in particular in PLWD [34]. For example, a recent systematic review of the validity of non-invasive sleep-measuring devices, aimed at assessing their future utility in dementia, was not able to identify any studies in people with mild cognitive impairment or Alzheimer’s Disease [35].

The ‘International Biomarkers Workshop on Wearables in Sleep and Circadian Science’ held at the 2018 SLEEP Meeting of the Associated Professional Sleep Societies identified that the main limitation of large-scale use of novel sleep and circadian wearables is the lack of validation against gold-standard measures [36]. The workshop formulated guidelines for validation and confirmed the following: PSG is the only valid reference for TST and sleep staging; PSG sleep records should be scored using current AASM guidelines; PSG sleep records should be double scored to minimise bias. Devices differ in the level at which they classify sleep-wake, from binary (sleep or wake) to four stages (wake (W), REM, light sleep (LS), deep sleep (DS)) to full AASM (W, stage 1 NREM (N1), stage 2 NREM (N2), stage 3 NREM (N3), REM) scoring. The ability of a device to over- or underestimate sleep and wake will depend on its sensitivity (ability to correctly classify sleep epochs), specificity (ability to correctly classify wake epochs), and accuracy (proportion of all epochs correctly detected) (reviewed in [36,37]). These factors will depend on the physiological variables and classification system used to determine sleep and wake. For example, according to traditional performance measures, actigraphy tends to have high sensitivity and accuracy but low specificity [38]. Despite this, actigraphy, when combined with a sleep diary, is considered a valuable tool for long-term monitoring of rest/activity patterns in clinical populations [39].

### 1.5. Our Approach to Technology Evaluation

Although it is crucial to evaluate the performance of a device against gold-standard measures, perhaps even more important is to assess its performance longitudinally in the real world where it will be used in both older adults and PLWD. Simultaneously, to increase our understanding of sleep and circadian rhythms in the real world and their interaction with disease processes, it is important to monitor relevant environmental variables such as light and temperature that may impact sleep or the circadian system but also aspects of waking function such as alertness, mood, and performance. Most validation studies are limited to healthy participants. Since co-morbidities are highly prevalent in PLWD, validation studies in, for example, cognitively intact older participants should use lenient inclusion/exclusion criteria to make the study more relevant to the intended use case. In addition, it is important to assess the acceptability, scalability and cost-effectiveness of any devices.

Here we describe our approach to evaluating novel wearable and contactless sleep, circadian, and environmental monitoring technology, in community-dwelling adults both at home and in the laboratory using multiple devices simultaneously against accepted standard measures. We first applied this approach in cognitively intact older adults to assess the performance and acceptability of a range of devices and have already published some of our findings [23,40,41,42,43]. Here we highlight some of our key findings in relation to the protocol design. We used our initial findings to select technology to assess in PLWD and their caregivers; this feasibility study is ongoing and so here we provide participant demographics to date, alongside example datasets.

#### 1.5.1. Selection of Participants

For our initial protocol in cognitively intact older adults, the eligibility criteria for participation in the protocol were designed to maximize the plausibility of relevance for home studies in the PLWD population. An essential criterion was that it was safe for the participant to participate in the study. Since co-morbidities are highly prevalent in PLWD, our initial validation study used lenient inclusion/exclusion criteria. Those with stable controlled medical conditions, with the exception of dementia, were included. Since most PLWD are older, the target population consisted of independently living, non-smoking, men and women aged 65–85 years. Some standard exclusion criteria were maintained. Participants had to consume ≤28 units of alcohol per week and be current non-smokers. By using these inclusion/exclusion criteria, our study population was relevant to PLWD and recruitment and, in addition, retention was very successful.

#### 1.5.2. Selection of Technology to Evaluate

We categorised and evaluated technology according to how it is used or what it is monitoring: (a) wearable devices that are placed on the body (e.g., wrist, head), (b) nearable/contactless devices that are placed near the individual (e.g., bedside or under the mattress) to detect physiological or behavioural signals, (c) environmental monitoring devices (e.g., light, temperature), (d) usable devices that the participant interacts with (e.g., electronic tablet for cognitive testing), (e) video monitoring that provides information about the individual and the environment.

The technology assessed in our completed and ongoing studies was selected based on the following criteria: (a) previous evaluation studies, (b) inclusion in comparable studies, (c) regulatory status, (d) cost, (e) potential acceptability to PLWD. We included both research-grade and consumer-directed devices. The technology selection also considered the potential burden on participants. For example, many wearables have limited battery life and may need regular charging during the course of the study. This not only will result in gaps in the data but also the potential for participants to forget to reapply the device after charging. In addition, some devices will need manual downloading whilst others will automatically upload data at the end of the recording to a cloud-based server (see Section 4.1.5 for further details on our approach). For all approaches, it is essential to ensure compliance with the general data protection regulations (GDPR).

We aimed to evaluate many devices simultaneously. An advantage of this approach is that the performance of a particular device can be compared to not only the standard methodology but also other devices. The number of devices we tested simultaneously took account of potential burden on participants and ensured that adequate signals could still be obtained when multiple devices were worn. Cognitively intact participants wore no more than four wrist devices (two per arm) at any one time, whereas in PLWD no more than two were worn. The technology used in each protocol is described in the Methods section. 

#### 1.5.3. Study Protocol

**Figure 1:**
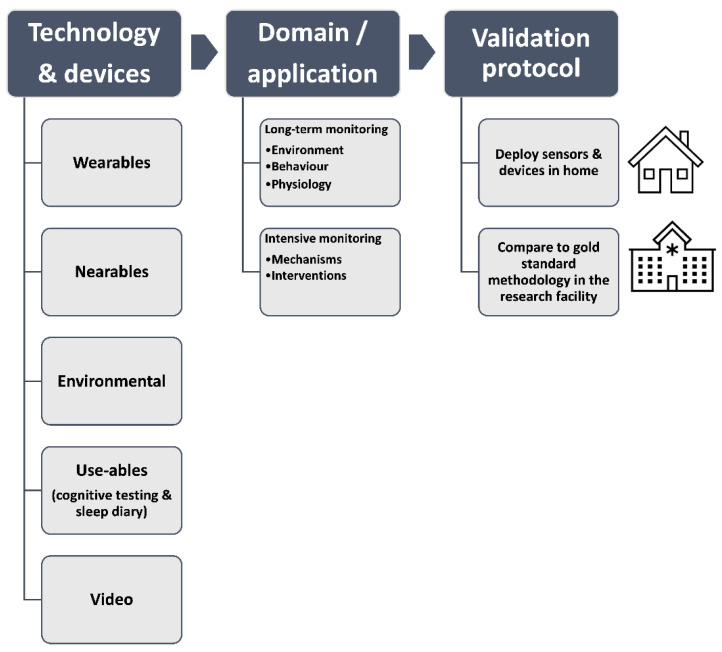
Overview of the concept of the protocol and categories of the devices included, the data and application domain, and the study design.

**Figure 2:**
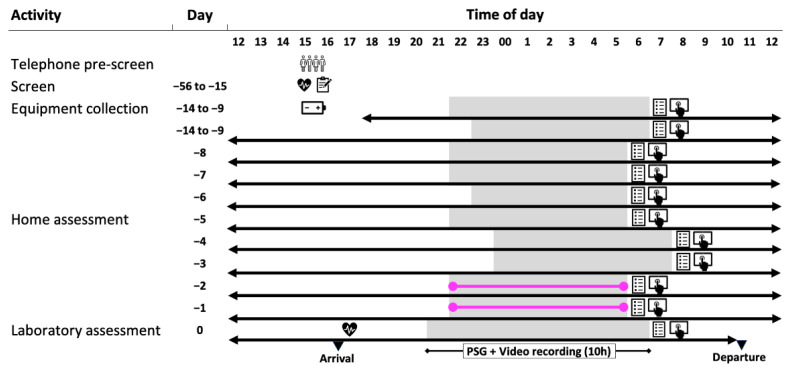
Schematic diagram of the protocol. The people symbol indicates when the telephone pre-screening assessment was conducted. The clipboard symbol indicates completion of questionnaires, the heart symbol when vital signs were measured, and the battery symbol indicates when participants were trained to use the technology. Grey bars indicate sleep periods and white bars indicate wake periods. The black horizontal line indicates the use of wearables and nearables throughout the at-home period. The pink horizontal lines indicate when an EEG device was used at home to measure sleep physiology. The symbol of a hand using an electronic tablet indicates completion of the cognitive test battery, and the questionnaire symbol indicates completion of the sleep diary.

Our protocol was designed to assess the performance and acceptability of a range of devices, firstly longitudinally at home (7–14 days) with actigraphy combined with a sleep diary as a reference point, and then in an overnight laboratory session with concurrent gold standard video-PSG. The AASM recommends that actigraphy is always accompanied by completion of sleep diaries to allow optimal interpretation of the actigraphic data [44], and that data are collected for a minimum of 72 h to 14 days [39]. The concept of the study is shown in Figure 1 and a schematic diagram of the protocol is shown in Figure 2.

Further details of the protocol can be found in the Methods section. Importantly, we provided ongoing support to the participants, including in-person training sessions (which were available 24/7) and, for PLWD, going to their homes to set up the technology. This approach assured compliance and a high level of data completeness.

## 2. Results

### 2.1. Recruitment and Participant Characterisation

For our initial protocol in cognitively intact older adults, we contacted *n* = 729 potentially eligible participants who were registered on the Surrey Clinical Research Facility (CRF) database. From these, *n* = 177 responded, *n* = 24 failed the initial telephone screen, and *n* = 46 were booked for screening visits, with the remaining *n* = 107 remaining on a waiting list. Of the *n* = 46 screened, *n* = 45 were deemed eligible for the study; of these, *n* = 3 withdrew consent and *n* = 6 were withdrawn due to the start of the COVID-19 pandemic and face to face research being suspended. We enrolled *n* = 36 participants into the study and 35 participants (14 female, 21 male) completed the study.

The demographics of these participants have previously been reported [23,25,40,41,43,44]. Briefly, they had a mean age of 70.8 ± 4.9 years (range: 65–83 years) and a mean BMI of 26.7 ± 4.7 kg/m^2^ (range: 20–40), which is representative of the UK population in this age bracket [45]. The participants had the following scores (mean ± SD) from the baseline questionnaires: (a) PSQI: 4.1 ± 2.1, (b) ESS: 3.6 ± 2.5, (c) ADL: 7.9 ± 0.2, (d) ICIQ: 1.0 ± 1.7, (e) S-MMSE: 28.7 ± 1.4.

Current co-morbid stable medical conditions were reported by 40% of participants. These included disorders of the following systems: endocrine, cardiovascular, gastrointestinal, respiratory, musculoskeletal, and ocular. In addition, 26% of the participants were taking prescribed medications including statins, ACE inhibitors, metformin, and calcium channel blockers.

Although according to the patients’ reported health status, only one participant was diagnosed with sleep apnoea, the PSG-based assessment revealed that 94% of participants had apnoea-hypopnea (AHI) scores indicative of sleep apnoea: 46% mild (AHI: 5–<15), 26% moderate (AHI: 15–<30), and 23% severe (AHI: ≥30).

For our feasibility study in PLWD and their study partners, to date we have enrolled seven control participants (65–85 years) (3 females, 4 males) who had a mean age of 67.0 ± 6.2 years (range 61–79 years) and a mean BMI of 26.4 ± 3.8 kg/m^2^ (range: 22–32.6). We have enrolled 11 PLWD (diagnosed with mild dementia/Alzheimer’s disease) of whom eight (4 females, 4 males) have completed the study and six of these had study partners (3 females, 3 males). The PLWD had a mean age of 74.8 ± 4.4 years (range 67–81 years), a mean BMI of 29.7 ± 7.6 kg/m^2^ (range: 20.7–42.1), and a mean s-MMSE score of 27 ± 1.6. The study partners had a mean age of 66.7 ± 14.8 years (range 44–80 years), a mean BMI of 28.9 ± 4.1 kg/m^2^ (range: 23.9–33.6), and a mean s-MMSE score of 28.8 ± 1.0. These participants also reported stable co-morbid medical conditions including type 2 diabetes, hypertension, arthritis, and hypothyroidism, and were taking concomitant medications.

### 2.2. Data Completeness

The 35 cognitively intact participants from our initial study collected data for 7–14 days at home which combined to a total of 397 days/nights. We recorded from 6–10 devices (number of nights per device depended upon participant and device), giving a total of 2748 device days/nights from all the devices combined with 95% data completeness. In the laboratory, we recorded 35 nights with 8–10 devices, giving 331 device nights, including PSG, and achieved 98% data completeness. As our feasibility study is ongoing, we cannot yet report data completeness.

### 2.3. Device Acceptability

Participants completed a single acceptability questionnaire on all of the technology used at home and in the laboratory. For each device, participants were asked to rate comfort (1—very uncomfortable to 7—very comfortable) and ease of use (1—very difficult to 7—very easy), and to record any problems. For our completed study in cognitively intact participants (*n* = 35), for all of the wearable devices combined, the participants rated comfort as 3.8 ± 1.9 and ease of use as 6.0 ± 1.2. For the nearable devices (*n* = 17 reported), participants rated comfort as 6.3 ± 1.2 and ease of use as 6.3 ± 1.1.

### 2.4. Examples of At-Home and In-Laboratory Recordings

One of the strengths of our approach, and of using multiple simultaneous devices, is that it is possible to obtain concurrent physiological, behavioural, and environmental signals both at home and in a laboratory setting. Here we show examples from individual participants of the datasets that were obtained.

**Figure 3:**
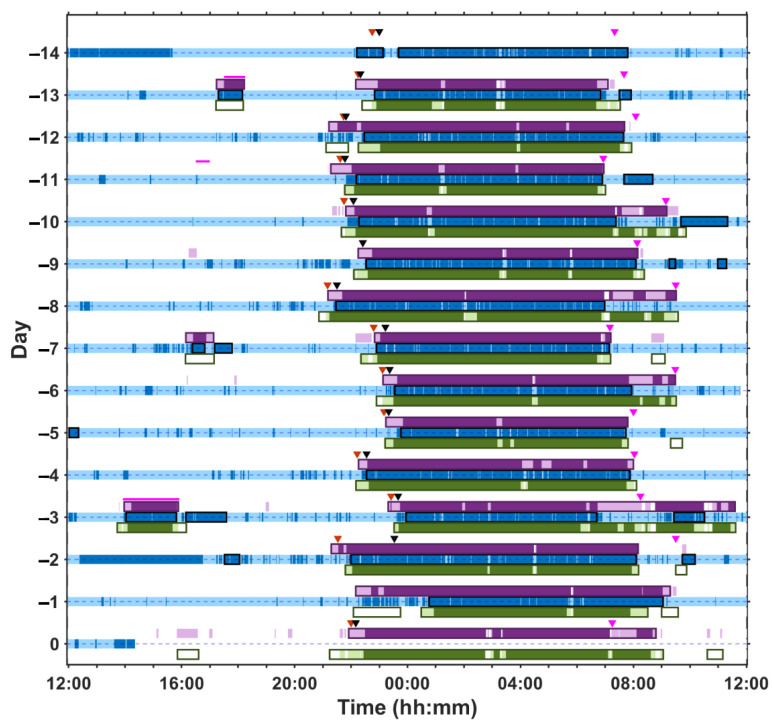
Multiple days of at-home recording (days −14 to −1) and a single overnight laboratory session (day 0) in a male participant in his 60s. The grey bars represent when the participant is out of bed and the purple and green bars represent when the participant is in bed as detected by two different ‘under the mattress’ nearable devices (Nearable 1 = Withings Sleep Analyser, WSA and Nearable 2 = EMFIT-QS, respectively). For Nearable 1, the light pink represents periods of wake, and the darker purple represents sleep; for Nearable 2, the light green represents periods of wake and the darker green represents sleep. The bed entry and bed exit times recorded on the sleep diary are represented by inverted grey and pink triangles, respectively, and the black triangle indicates estimated sleep onset according to the sleep diary. The horizontal magenta lines represent nap times recorded on the sleep diary. The blue bars represent wrist worn actigraphy with dark blue indicating sleep and light blue representing wake.

Figure 3 shows exemplar data from a cognitively intact male participant in his 60s who had moderate sleep apnoea (AHI = 24.1) and was living with controlled type 2 diabetes. The raster plot includes 14 consecutive days of home recording followed by the overnight in-laboratory session, simultaneously using two contactless, nearable technologies (WSA and EMFIT) and a wrist worn actigraphy device (actigraphy was not used in the laboratory) with completion of a subjective sleep diary. The raster plot provides a measure of sleep behaviour and shows the day-to-day variation in sleep timing. The two under-mattress, contactless devices accurately detect bed presence as demonstrated by their concordance with the information captured in the sleep diary [41]. These devices can also capture daytime naps taken in bed and generate automated sleep summaries, without using information from a sleep diary (boxed regions in the raster).

**Figure 4:**
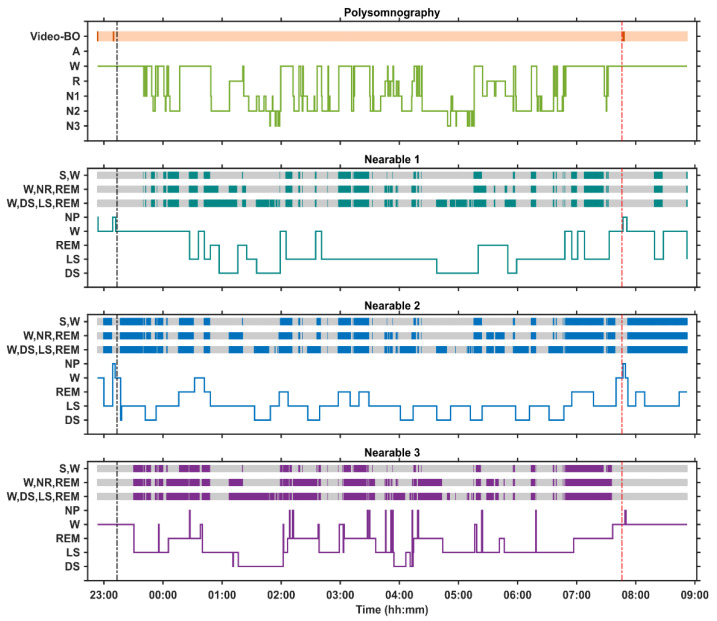
Hypnograms from a 10-h in-bed period in a laboratory environment in a single participant with simultaneous polysomnography, including video, and three nearable devices (Nearable 1 = Withings Sleep Analyser, Nearable 2 = EMFIT-QS, Nearable 3 = Somnofy) The black vertical dotted line depicts Lights Off and the red vertical dotted line depicts Lights On. The orange horizontal bar represents bed occupancy according to the video, with darker lines indicating when the participant left the bed. For each nearable, the discrepancy between the nearable and the PSG determined sleep is depicted at three different levels of sleep stage classification: (1) wake vs. sleep, (2) wake vs. NREM vs. REM, (3) wake vs. deep sleep vs. light sleep vs. REM. The darker coloured region indicates epochs of discrepancy. BO = bed occupancy, S = Sleep, W = wake, NR = NREM, NP = not present, REM = rapid eye movement sleep, LS = light sleep, DS = deep sleep.

Figure 4 provides an example of data captured in the laboratory session where polysomnography was recorded for 10 h with simultaneous use of three nearable devices. We implemented a 10 h time-in-bed period to induce a lower sleep efficiency, which allows for a better evaluation of the device’s ability to correctly classify both sleep and wakefulness. For each nearable, discrepancy between the device and manually scored PSG is indicated with darker coloured bands at three different levels of sleep stage classification: (1) wake vs. sleep, (2) wake vs. NREM vs. REM, (3) wake vs. deep sleep vs. light sleep vs. REM. Some of the devices already ‘detected’ sleep before lights-off or after lights-on. Please note that in many laboratory evaluation studies, the device performance is assessed only during the lights-off phase. Since in the real world, the lights-off-lights-on information is in most cases not available, it is important to evaluate their performance over the entire in-bed period, and not just during the lights-off period. For all three nearable devices, it can be seen that as the resolution of the sleep staging increases, so do the number of discordant epochs. Thus, while the nearable devices might be able to distinguish to some extent between wake and sleep, this is not accurate as they identify epochs of sleep before lights-off/after lights-on when the participant is awake. They also cannot accurately distinguish between different stages of sleep.

**Figure 5:**
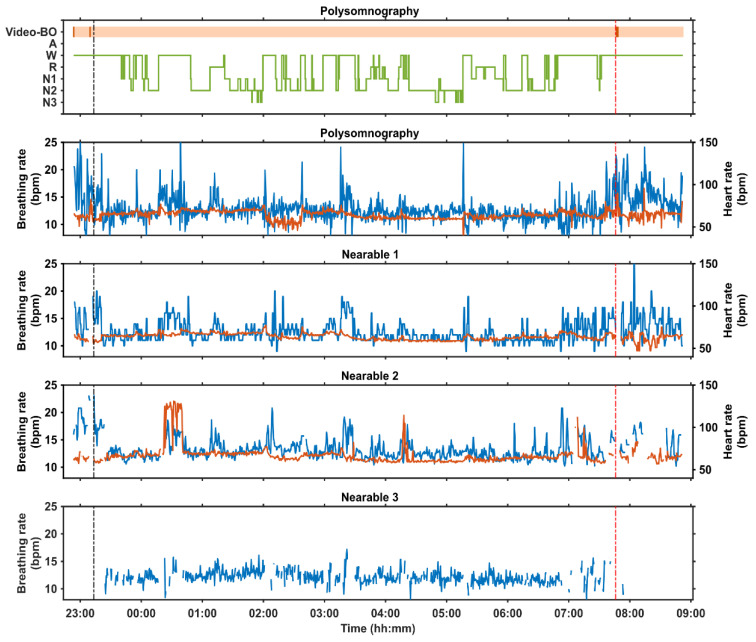
Physiological measures during a 10 h in-bed period in a laboratory environment in a single participant with simultaneous recordings of polysomnography, including video, and from three nearable devices. The black vertical dotted line depicts Lights Off and the red vertical dotted line depicts Lights On. The orange horizontal bar represents bed occupancy according to the video, with darker lines indicating when the participant left the bed. For each nearable device, blue lines represent breathing rate and red lines represent heart rate. Within the PSG hypnogram, BO = bed occupancy, A = artefact, W = wake, R = REM sleep, N1 = stage 1 NREM sleep, N2 = stage 2 NREM sleep, N3 = stage 3 NREM sleep. Devices: Nearable 1 = Withings Sleep Analyser, Nearable 2 = EMFIT QS, Nearable 3 = Somnofy.

Figure 5 provides an example of heart rate and breathing rate captured from PSG and three nearable devices from a single participant during a 10 h period in bed in the laboratory. The pattern observed in the vital signs with Nearable 1 matches that detected by the gold-standard PSG signals, whereas Nearable 2 recorded some spikes in heart rate that are not seen in the PSG or Nearable 1. Nearable 3 only record breathing rate, and the signal was consistent across the course of the night.

**Figure 6:**
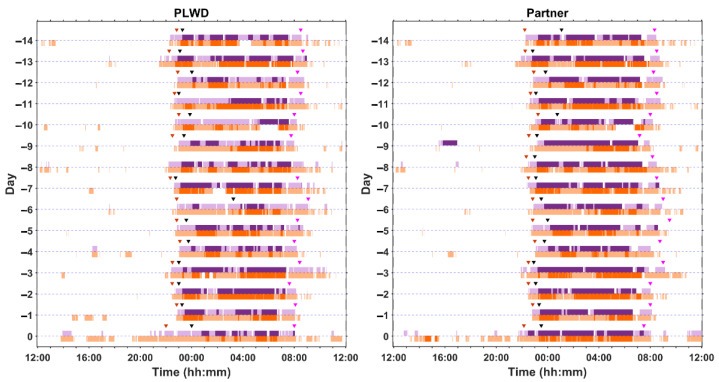
Multiple days of at-home recording (days −14 to −1) and a single overnight laboratory session (day 0) from a PLWD and their partner who share a bed. The white bars represent when the participant is out of bed and the purple and red bars represent when the participant is in bed as detected by two different nearable devices (Nearable 1 = Withings Sleep Analyser (WSA) and Nearable 2 = Somnofy, respectively). The purple bars represent an under-mattress sensor and the red bars a bedside sensor; for both, the darker shading indicates sleep and the light shading indicates wake as determined by the devices. The bed entry and bed exit times recorded on the sleep diary are represented by inverted grey and pink triangles, respectively, and the black triangle indicates estimated sleep onset according to the sleep diary.

Figure 6 shows multiple consecutive days of recording in a woman living with dementia in her 70s and her partner, a man in his 80s, who share a bed. The PLWD’s sleep is fragmented and varies from day to day in terms of duration and timing. The partner also experiences nights of discontinuous sleep.

**Figure 7:**
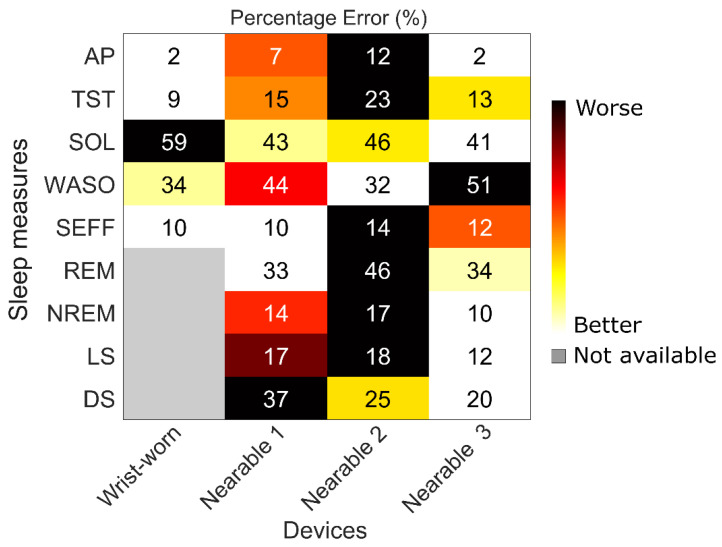
Percentage error (%) in the all-night sleep measures estimates determined by four devices used in the study protocol. (Wrist-worn wearable = Actiwatch spectrum; Nearable 1 = Withings Sleep Analyser, Nearable 2 = EMFIT QS, Nearable 3 = Somnofy). (Figure adapted from: Ravindran, G.K.K.; della Monica, C.; Atzori, G.; Lambert, D.; Hassanin, H.; Revell, V.; Dijk, D.-J. Three Contactless Sleep Technologies Compared to Actigraphy and Polysomnography in a Heterogenous Group of Older Men and Women in a Model of Mild Sleep Disturbance: A Sleep Laboratory Study. JMIR mHealth and uHealth 2023, 25/08/2023:46338—(forth-coming/in press). URL: https://mhealth.jmir.org/2023/1/e46338. Licensed under Creative Commons Attribution cc-by 4.0) [40].

Figure 7 provides a visual representation of the difference in the device performance for standard sleep measures and allows effective comparison. For example, if sleep onset latency is of primary importance to a protocol, use of Nearable 3 would be recommended since it provides a more accurate estimate compared to other devices, while for sleep efficiency estimation, the use of a wrist-worn actiwatch would provide similar or better accuracy compared to a nearable.

**Figure 8:**
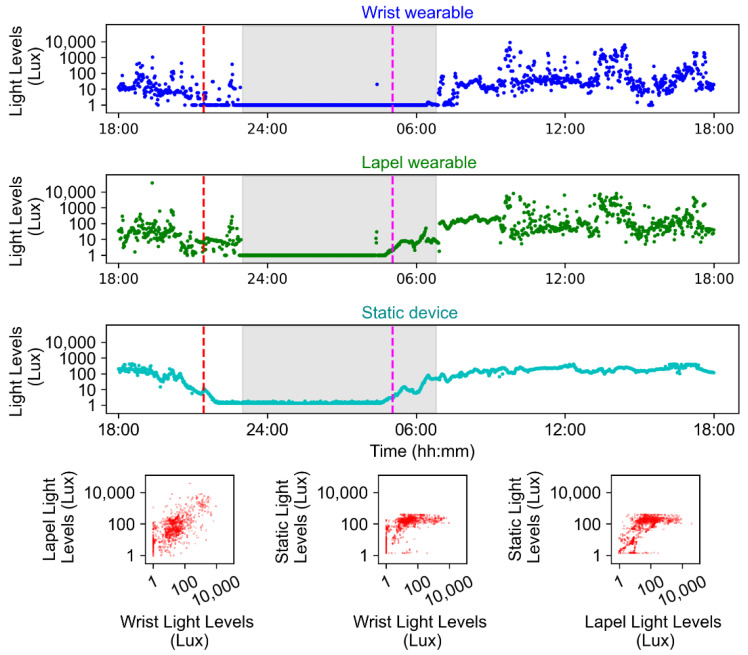
Light exposure data (plotted on a Log scale) measured over a 24 h period for one participant using three devices: a wrist-worn wearable (blue), a collarbone/lapel worn wearable (green), and a static room device (black) (where the participant spends the majority of their time). Grey shaded areas indicate sleep periods reported in the sleep diary, the vertical red dashed line is sunset, and the vertical pink dashed line is sunrise. The scatter plots indicate the relationship between the light measures obtained by the different devices.

Figure 8 is an example of light exposure data for a 24 h period for an individual from two worn devices, as well as the light levels in the room in their house in which they spent the majority of their time. For the 17 participants who wore both the actiwatch and HOBO, there was a significant correlation between the values measured by the two devices (r = 0.36, *p* < 0.001). For this individual, their morning light exposure was up to 10,000 lux and exceeded the light levels in their home, suggesting they were outside. Their afternoon light exposure varied between 10 and 1000 lux but is generally lower than the light levels measured in their house, suggesting they were indoors. Their evening light exposure was quite low and generally <100 lux.

## 3. Discussion

We have presented our approach for evaluating multiple, concurrent sleep/circadian monitoring technologies both at home and in the lab against accepted standard measures in older people and PLWD. This approach is rather different from published approaches in terms of the population enrolled, the number of devices evaluated simultaneously in one individual, the use of home and lab assessments, and the analysis intervals used for performance evaluation. In particular, our inclusion of a heterogeneous population for this age range is in contrast to the stringent criteria, in relation to health conditions and medication, applied for most clinical trials. Our high level of data completeness (95–98%) and participant retention (97%) is an indication of the success of our approach. We evaluated device performance (Section 4.1.6, Section 4.1.7 and Section 4.1.8) over an extended period in bed to ensure that we included both sleep and quiet wakefulness. This ensured that we can determine how well the device performs in people with disturbed sleep and poor sleep efficiency, which is a more relevant use-case for many health conditions. We have previously reported some of our findings [40].

The majority of previous evaluation studies have taken the approach of either validating a single device against gold standard, or assessing multiple devices (but not simultaneously) in the same individual. For example, Chinoy and colleagues took a similar approach to us in simultaneously comparing seven consumer sleep tracking devices (wearable and contactless) in young participants at home and in the laboratory, but participants only used a subset of the devices [37]. A strength of our design is that participants utilised multiple devices simultaneously to maximise evaluations and comparisons.

The predominant inclusion of young and healthy individuals in published device evaluation studies limits the applications of the findings [37,46,47,48]. In addition, previous studies only evaluated the performance of a device over a lights-off period selected by the participants, meaning sleep efficiency is high and so it is not possible to evaluate the ability of the device to discriminate quiet wake (e.g., [46,47]). Indeed, in a young healthy population, it was demonstrated that the performance of both wearable and contactless devices worsened on a night of sleep disruption (when sleep efficiency is poor) compared to an undisturbed night in the laboratory [37].

The value of assessing the device in the population in which it will be used was highlighted by three recent studies. An assessment of the EMFIT-QS mattress sensor in participants from a sleep disorders centre with a BMI of 33.8 ± 8.3 kg/m^2^ (mean ± SD; range 21.4–46.6) revealed that this device overestimated TST and underestimated WASO [49]. However, the authors noted that the performance actually worsened in those with a high apnoea-hypopnea index (AHI) and more fragmented sleep, but that estimations of TST actually improved in participants with increased weight and BMI, suggesting that the device performs better with bigger movements from heavier individuals [47]. The Withings Sleep Mattress Analyser (WSA) was assessed in participants with suspected sleep apnoea, and the device similarly overestimated sleep and underestimated wake, but could accurately detect moderate to severe sleep apnoea (AHI was 31.2 ± 25 and 32.8 ± 29.9 with PSG and WSA, respectively), highlighting its diagnostic and monitoring value [50]. Finally, 293 cognitively normal and mildly impaired older adults were monitored for up to six nights at home using single-channel EEG (scEEG), actigraphy, and sleep diaries [51]. Estimates of TST showed the greatest agreement across all methods, particularly for cognitively intact adults, but the agreement between actigraphy and scEEG decreased in those with mild cognitive impairment and biomarker evidence of Alzheimer’s Disease. These studies and our approach emphasize that it is crucial that the device is evaluated in a relevant population for its intended use.

The devices we are testing do have potential for long-term use in the home environment. The Withings Sleep Analyser has been deployed in PLWD where variation in night-time behaviour and physiology was shown to relate to disease progression, comorbid illnesses, and changes in medication [52].

The approach we describe here could be applied to evaluate the performance and acceptability of any novel sleep or circadian monitoring device in any population. Combining information from multiple devices can assist with interpreting sleep-wake behaviour as well as allowing their performance to be cross-validated. For example, the AWS provides information about activity levels but not about whether the participant is in bed; however, when the AWS data is viewed in conjunction with the Withings Mattress data, it is possible to identify when the participant has left the bed rather than just being restless in the bed. This is particularly relevant to PLWD whose nocturnal wandering is a major reason for them to be moved from their home into a care home.

One challenge of the protocol was that many participants had not used smart technology previously, and we required participants to complete a number of procedures independently, which caused some PLWD to express concern about whether they would remember. Thorough training sessions, provision of comprehensive written instructions, the role of the study partner for PLWD, and frequent contact between researchers and participants, ensured that they felt supported and able to carry out all study procedures. We note that the PLWD included in our study were experiencing mild Alzheimer’s. The performance and acceptability of devices in more advanced stages of Alzheimer’s remains to be addressed. Contactless monitoring devices with very low or ‘nil’ user burden, such as under-the-mattress devices, are more likely to be useful in these populations.

In conclusion, our protocol allows multiple sleep/circadian/environmental technologies to be assessed simultaneously in an individual both at-home and in the laboratory. Our approach was successful in terms of data quality, data completeness, and gaining an understanding of device acceptability. The protocol was conducted in both cognitively intact older adults and PLWD to provide a comprehensive picture of an individual’s behaviour, physiology, and environment.

## 4. Materials and Methods

### 4.1. Study Design

#### 4.1.1. Study Conduct

The protocols were guided by the principles of Good Clinical Practice. All participants were compensated for their time and inconvenience. Within the participant information sheet, it was clearly stated that all personal data were handled in accordance with the general data protection regulations (GDPR) and the UK Data Protection Act 2018. In addition, it was explained that anonymised non-personal data may be transferred to the manufacturers of the devices being tested if they need to process the data. The manufacturer can only access anonymised data that details the serial number of the device, the date of recording, and the signals recorded on that specific date. The participants consented to the manufacturers using their anonymised data in the continual assessment and improvement of the performance of the device.

#### 4.1.2. Participants

For our protocol in PLWD and their caregivers, the inclusion/exclusion criteria for PLWD included the following: age range of 50–85 years, a confirmed diagnosis of prodromal or mild Alzheimer’s disease, an S-MMSE (Standardised Mini Mental State Examination [53]) score > 23, living in the community, and, if taking medication for dementia, being on a stable dose for at least three months prior to recruitment. Individuals who had an unstable mental state, severe sensory impairment, active suicidal ideation, or being treated for terminal illness were excluded. PLWD could participate in the study by themselves, or their carer/family support/friend could also enrol as a ‘study partner’ participant. These study partners had to be > 18 years of age, have an S-MMSE score > 27, and must have known the PLWD for at least six months and be able to support them in their participation. Study partners completed the same procedures as the PLWD.

Cognitively intact older adults were recruited via our Clinical Research Facility database, where potential participants have registered and consented to be contacted about ongoing research. PLWD and their study partners are recruited in collaboration with local NHS trusts via memory services. All participants underwent an initial telephone health screening and subsequent in-person screening visit to determine their eligibility to take part in the study.

At the screening visit for cognitively intact older adults, following informed consent, participants completed a range of assessments including measurement of height, weight, and vital signs (body temperature, heart rate, respiration rate, and blood pressure), self-reported medical history, and completion of baseline questionnaires: Epworth Sleepiness Scale (ESS) [54] (>10 indicates excessive daytime sleepiness), Pittsburgh Sleep Quality Index (PSQI) [55] (>5 indicates a sleep disorder), Activities of Daily Living Questionnaire (ADL) [56], and International Consultation on Incontinence Questionnaire—Urinary Incontinence (ICIQ-UI) [57].

At the screening visit for PLWD (and study partners, where applicable), and following informed consent, the participants completed standard assessment tools which are frequently used in this population (i.e., sMMSE [56], Hospital Anxiety and Depression Scale (HADS) [58], quality of life in Alzheimer’s Disease (QoL-AD) [59], as well as the PSQI [55], and medical history questionnaire). In addition, vital signs were recorded as well as height, weight, and BMI. PLWD and their study partners also completed additional questionnaires either at this visit or during their overnight sessions: ESS, ADL, ICIQ, National adult reading test (NART) [60], Berlin questionnaire to assess for sleep apnoea [61], Horne-Ostberg questionnaire [62] to assess time of day preference, and their education level was documented. For all participants, their general practitioner was informed of their participation.

#### 4.1.3. Longitudinal Monitoring At-Home

Participants were provided with a range of technology to use in their home to monitor their sleep/wake patterns and environmental light exposure (see Table 1 for list of devices and variables measured). The technology was either installed by the participants themselves or researchers went to the participants’ homes to assist them. The wrist-worn/collarbone-worn devices were worn continually and participants were requested to complete a log whenever they removed them to record times and reason for removal. The EEG wearables were only used for one or two nights and the nearables were left in situ throughout. Participants were requested to complete a modified version of the Consensus Sleep Diary-M [63] (electronically or on paper) on a daily basis to record subjective information about their sleep patterns, sleep quality, daytime napping, as well as alcohol and caffeine consumption. In addition to the standard questions, participants were asked to provide further details about their daytime naps (what time, duration, where and why they napped) and nocturnal awakenings (for each awakening, what time they awoke, how long it took to fall asleep, if they left the bed, and if so, at what time). Participants were requested to complete cognitive assessments one to two hours after waking each day on an electronic tablet.

#### 4.1.4. Overnight Laboratory Session

The session was ~24 h in duration and participants were required to arrive in the afternoon and remain at the Research Centre, which hosts the UKDRI clinical research facility at Surrey, until the following day. Upon arrival, participants’ vital signs were measured, and continued eligibility assessed. The devices that were used at home were downloaded, reset, and returned to the participants with any additional devices only used in the laboratory. During their stay, participants’ gait and postural stability were assessed using video and radar technology.

During the laboratory session, participants had an indwelling cannula sited for collection of regular blood samples at three-hourly intervals (including overnight) for 24 h to assess time of day variation in biomarkers. The samples were processed and were analysed for levels of melatonin, as a gold-standard marker of the circadian clock, as well as biomarkers of dementia e.g., neurofilament light (NfL), phosphorylated tau (p-tau), amyloid-beta (AB40 and AB42; e.g., [65,66,67]). In addition, participants collected urine for 24 h in four-hourly intervals (eight hours overnight) for measurement of aMT6s.

Following dinner, participants were equipped with all the electrodes and sensors required for a clinical video-polysomnographic (PSG) recording using AASM compliant equipment and montage. The PSG equipment was the Somnomedics SomnoHD system with Domino software (v 3.0.0.6, sampled at 256 Hz; SOMNOmedics GmbHTM, Randersacker, Germany), and we used an American Academy of Sleep Medicine (AASM) standard adult montage. Participants could also have a wearable EEG device fitted for concurrent EEG recording, and contactless sensors were positioned for overnight recordings. Prior to the start of the PSG recording, participants were asked to lie on the bed in different supine poses (prone, supine, right, left, seated) and recordings were made with video and radar technology to assess the ability of the radar technology to assess physiology in different poses.

The protocol takes advantage of the ‘first night effect’ and an extended period in bed to create a model for mildly disturbed sleep [68]. Participants were required to be in bed for a 10 h recording period that was determined on the basis of their habitual time in bed period (HTiBP). For example, for HTiBP < 8 h, the recording period started one hour earlier than habitual bedtime; for HTiBP > 10 h, the recording started at habitual bedtime. For those with 8 h ≤ HTiBP ≤ 10 h the recording start time was determined as Habitual Bedtime—[0.5 × (10 − HTiBP)]. This extended period in bed was used to ensure that the recordings included periods of quiet, recumbent wake to determine if the devices could distinguish quiet wake from sleep. Participants selected their own lights off/on times and around these times were permitted to conduct quiet, sedentary activities, e.g., watching movies, reading. Overnight recordings were performed in individual, environmentally controlled bedrooms or in our bespoke bedroom facility that has double occupancy or adjacent room access for PLWD and their study partners.

Upon awakening, participants were requested to complete their sleep diary and cognitive test battery as well as a questionnaire about device acceptability. Prior to discharge, vital signs were taken and, for cognitively intact older adults, the S-MMSE was administered.

#### 4.1.5. Device and Data Management

**Figure 9:**
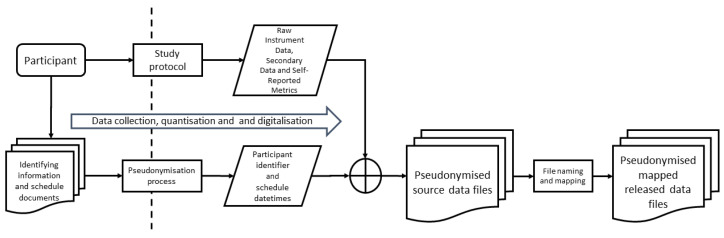
Overview of data acquisition and data management. The two sides of the dotted line on the diagram represent the participant, environment, and paper documentation during the time of the study and the collection of digital representations of these, respectively, from left to right.

The flow of data acquisition and data management is shown in Figure 9.

##### Device Allocation

Eligible participants were enrolled into the study and had a set of devices allocated to them. All devices and systems had unique identifiers, with a one-to-one allocation as follows: (a) device code to participant for wearable/contactless devices used at home or in the lab, and (b) device to location to participant for devices installed in the laboratory (e.g., floor sensor data is recorded for a specific lab room, and the room is allocated to the participant). All devices were mapped to an operation schedule, which means that data were collected during each 24 h period from 12 noon to 12 noon the following day, unless the device had continuous recording.

##### Device Set-Up and Synchronisation

To be able to directly compare the performance of different devices, it is essential that they are time synchronised. All network device clocks were synchronised to a Network Time Protocol (NTP) server. Commercial standalone systems were synchronised through the respective software applications used to set up the device recordings.

##### Data Acquisition

The devices used were either battery-powered logging devices or were directly connected to power, and either stored data locally or were wi-fi enabled and transmitted data to the secure cloud servers of the manufacturers. Participants were provided with an independent Wi-Fi 4G gateway for device connection.

Upon arrival at the laboratory, all devices were collected from participants for the following: (a) download of data and confirmation of power levels for battery powered devices, (b) reconfiguration of connected devices to connect to local Wi-Fi connections. Devices to be used during the laboratory session were returned to the participants.

At the end of the lab session, all logging devices were connected to the relevant secure system and source data files extracted and moved to a location based on the participant, day(s), and device for that data. For the online server-based systems, these were synchronised, and data were then extracted and placed into the relevant source data file system.

##### Data Mapping and File Name Convention

All data recorded as a source file, or in a source system, were mapped to a named file and location based on study-specific parameters, e.g., Study Name (required), Device code (required), Test/Data/Measure (Optional), Participant or group (required), Visit (required), Study day/night (required). Access to this Research Data Store (RDS) was strictly controlled in accordance with Information Governance procedures for the specific use of the study protocol owners for analysis.

#### 4.1.6. Data Processing and Analysis

To evaluate the accuracy and reliability of the focus technology (devices being evaluated) in measuring sleep, we compared it to a standard reference technology. For the at-home recordings, the comparative standard measure against which all other technologies were evaluated was the combination of the Actiwatch-spectrum (AWS) and Consensus Sleep Diary (cognitively intact adults), or AX3 and Consensus Sleep Diary (PLWD and study partners). For the in-laboratory session, the PSG is considered the gold-standard measure [21,39,69] for all participants. The PSG recordings were scored in 30 s epochs (in accordance with AASM guidelines) by two independent scorers, and a consensus hypnogram was generated. AHI was determined using the AASM criteria for scoring apnoea/hypopneas where there is > 3% drop in oxygen saturation and/or an arousal.

**Figure 10:**
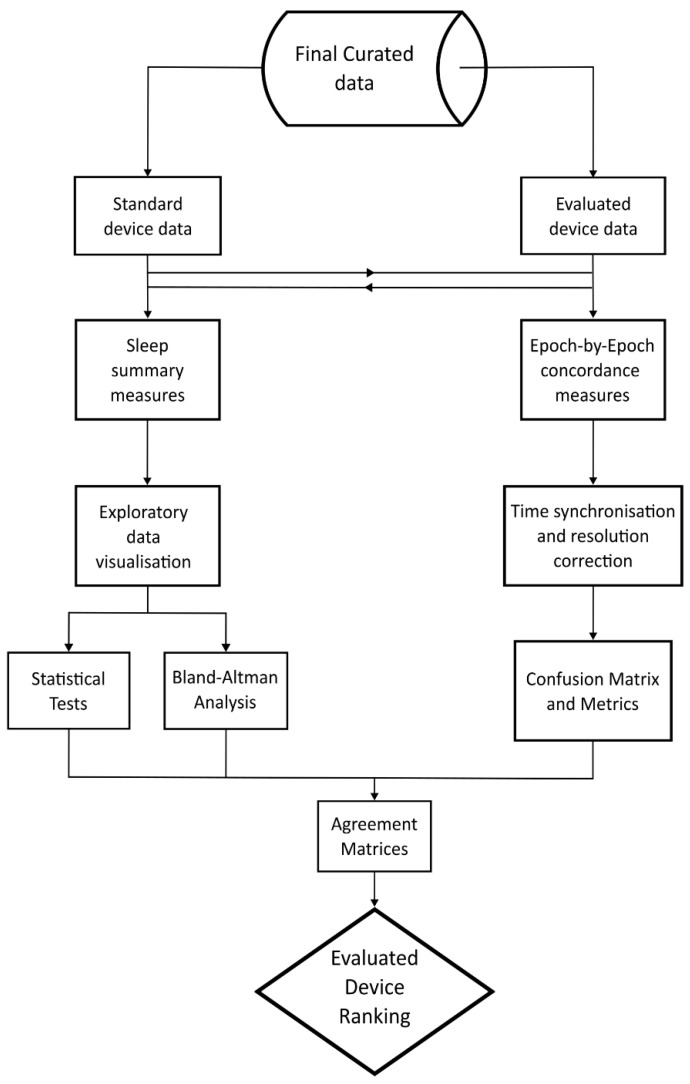
Overview of process for evaluating consumer-grade technology against standard device.

The focus technology was evaluated against the standard reference technology (AWS + sleep diary or PSG) for its ability to estimate sleep summary measures (e.g., TST, SOL). In addition, in the laboratory, the focus technology was assessed for its epoch-by-epoch (EBE) concordance with the PSG hypnogram. Sleep summary measures were obtained from processing data using local individual proprietary software for battery logging devices, or via raw signals being uploaded to a cloud-based server for scoring by a proprietary machine-learning based algorithm. Our approach to data analysis is depicted in Figure 10.

#### 4.1.7. Sleep Summary Measures

The sleep summary measures can be grouped into two categories: (1) sleep/wake measures, e.g., TST, SOL, WASO, and SE, and (2) sleep stage duration measures. The sleep stage duration measures vary depending on the level at which the focus device classifies sleep-wake, i.e., binary, four stages, or full AASM. The interval over which the sleep summary measures were calculated (analysis period) was either automatically set by the device algorithm, or could be manually set using either the sleep diary reported times of attempted sleep and final awakening (standard for at-home recording) or the lights off period or total recording period (standard for in-laboratory recording) [21,39]. The analysis period chosen can have a substantial impact on the summary measures calculated and the performance of the device when compared to home/laboratory standards [40,41].

All of the focus technologies generated summary measures automatically using the device algorithm-determined analysis period. The primary analysis was performed using these automatic summary measure estimates. However, for completeness, the summary measures could also be manually calculated.

A number of data visualisations (scatter plots, box plots, QQ plots, etc.) were performed to check the distribution of the data and for the presence of outliers. Further statistical tests were performed to check the normality of the data. For the agreement estimation, Bland-Altman analysis was performed, and bias, limits of agreement, and minimum detectable change were estimated. Other metrics that could be computed included, for example, Pearson’s correlation, consistency intraclass class correlation (ICC), effect size (Cohen’s D), and mean absolute percentage error (MAPE) [70,71]. To rank the devices, the agreement matrices containing the sleep measure accuracy metrics were created.

#### 4.1.8. Epoch-By-Epoch (EBE) Concordance

At-home recordings: the resolution of the focus technology’s hypnogram was reduced to binary sleep/wake classification to match the AWS which was used as the comparative standard measure. The analysis window was set between 18:00 h and 12:00 h, and all common periods of sleep/wake timeseries were evaluated.

In-laboratory recordings: the PSG hypnogram resolution was reduced to match the levels of sleep stage output by the device (e.g., N1 + N2 = light sleep (LS) and N3 = deep sleep (DS)) to allow direct comparison. Only valid pairs of epochs between PSG and device were used for the concordance analysis. The analysis window was set as the total recording period (~10 h). The EBE concordance metrics for the devices were estimated from the confusion matrices constructed. The concordance metrics used for the analysis of all the different sleep stage levels included sensitivity, specificity, accuracy, Matthew’s correlation coefficient (MCC), and F1 score. Similar to the sleep summary measures analysis, an agreement matrix for EBE concordance was created using MCC. MCC is preferred to other concordance metrics since it accounts for class imbalance commonly encountered in hypnogram data and is a better alternative to the metrics such as kappa or its variants [72]. The final device ranking was created using the summary sleep measures and EBE agreement matrix. Furthermore, the effect of participant characteristics such as age, sex, BMI, AHI, and other confounding factors on the device accuracy and reliability were also explored.

#### 4.1.9. Environmental Measures

Characterising the environment, in particular light, is crucial to understanding sleep/circadian physiology in the real world and in different disease states [73,74]. In the current studies, light exposure patterns were assessed by both static and worn devices which recorded lux values at one-minute intervals. One wearable (which measures white, red, green and blue light) was worn on the wrist and one was clipped on clothing near the collarbone; the static device was placed in the room of the home where the participant spent the majority of their time. During data visualisation, imputation was performed for any periods during which the participants were awake but the measured light levels were zero lux, which could be due to the sensors being accidentally covered. The imputation consisted of replacing these zero values with the median value from the preceding and succeeding 30 min. This was calculated across all available days of data for each participant separately. The consistency of measurements between devices was assessed by performing correlations between the lux values obtained by the wrist-worn and collarbone-worn devices.

#### 4.1.10. Quality Assurance and Mitigating Issues Troubleshooting

To maximise data completeness and quality, during the study, data acquisition from wi-fi enabled devices was monitored daily and participants could be contacted if any issues arise. Participants were also able to contact the researchers 24/7 with any issues or concerns. Some potential issues that may arise and their mitigations are presented in Table 2.

#### 4.1.11. Expertise Needed to Implement the Protocol

These studies require a team of trained personnel to ensure participant safety and well-being as well as data quality and integrity, including troubleshooting issues with devices. This level of support is required from the point of consent, throughout the at-home data collection, and for the overnight laboratory session. In addition, specialised and competent staff are required for collecting blood samples, PSG instrumentation, PSG recordings, PSG scoring, and data analysis.

## Figures and Tables

**Table 1:** Commercially available and research-grade devices used in the study protocols.

Location	At Home/in Lab	Study—Cognitively Intact	Study—PLWD & Study Partner
Wearables			
Wrist	At home & in Lab	Actiwatch Spectrum (Philips Respironics, Bend, OR, USA): activity; sleep estimates; white, red, green, and blue light. Withings Steel watch (Withings, Issy-les-Moulineaux, France): activity, sleep estimates, heart rate. AX3 (Axivity, Newcastle upon Tyne, UK): activity, white light. Apple watch (Apple, Cupertino, CA, USA): activity, sleep estimates, heart rate.	Withings ScanWatch (Withings, Issy-les-Moulineaux, France): activity, sleep estimates, heart rate. AX3 or AX6 (Axivity, Newcastle upon Tyne, UK): activity, white light.
	In Lab only	E4 (Empatica, Cambridge, MA, USA): activity, wrist temperature, heart rate.	
Head	At home & in Lab	Dreem Headband 2 (Dreem, Paris, France): EEG sleep, position, activity.	Dreem Headband 2 (Dreem, Paris, France): EEG sleep, position, activity.
	In Lab only	Marvels ear-EEG sensor prototype developed by Imperial College (Imperial College, London, UK)	Marvels ear-EEG sensor prototype developed by Imperial College (Imperial College, London, UK) Somnomedics Home Sleep Test (SOMNOmedics GmbHTM, Randersacker, Germany): EEG sleep.
Nearables			
Under mattress	At home & in Lab	Withings Sleep Mattress Analyser (Withings, Issy-les-Moulineaux, France): sleep, breathing rate, heart rate, activity, bed occupancy. EMFIT QS (EMFIT Limited, Vaajakoski, Finland): sleep, breathing rate, heart rate, activity, bed occupancy.	Withings Sleep Mattress Analyser (Withings, Issy-les-Moulineaux, France): sleep, breathing rate, heart rate, activity, bed occupancy.
	In Lab only	N.A.	N.A.
Nightstand	At home & in Lab	N.A.	Somnofy (VitalThings AS, Tønsberg, Norway): sleep, breathing rate, bed occupancy.
	In Lab only	Somnofy (VitalThings AS, Tønsberg, Norway): sleep, breathing rate, bed occupancy. Tiresias networked radar system prototype (Imperial College, London, UK) [64]: sleep, breathing rate, bed occupancy.	Tiresias networked radar system prototype (Imperial College, London, UK) [64]: sleep, breathing rate, bed occupancy.
Environmental			
One worn at level of collarbone when awake & one hung in living space	At home & in Lab	HOBO (Tempcon, Ford, UK): white light, temperature.	HOBO (Tempcon, Ford, UK): white light, temperature.
	In Lab only	N.A.	N.A.
Usables			
N.A.	At home & in Lab	Electronic tablet with Cognitron and sleep diary.	Electronic tablet with Cognitron and sleep diary.
	In Lab only	N.A.	N.A.
Video			
Sleep Lab	At home & in Lab	N.A.	N.A.
	In Lab only	Somnomedics video camera (SOMNOmedics GmbHTM, Randersacker, Germany).	Somnomedics video camera (SOMNOmedics GmbHTM, Randersacker, Germany).

**Table 2:** Device evaluation studies: potential issues and mitigations.

Potential Issue	Mitigation
Device synchronization: Devices may not be time synchronized if they were set up/downloaded/analysed on different systems. This could be due to the fact that some devices will use timestamps on local machines whereas others use UTC. This has previously been identified as being critical for epoch-by-epoch analysis [36].	Possible solutions could be using a physiological signal, e.g., eye blinks or moving the wrist, as a synchronizing signal for cross correlation.
Missing data: This could occur due to equipment malfunction, user error, data signal loss, data storage insufficiency, or user error (e.g., wearing the device incorrectly, not using the device when required, forgetting to update apps, forgetting to enter data, unplugging or obstructing nearable devices).	Potential mitigations include: (a) ensure participants are thoroughly trained in the use of all equipment and provide instructions to take home, (b) where possible, remotely monitor data acquisition and follow up if needed, (c) test all equipment before use.

## Data Availability

All data can be made available upon reasonable request to C.d.M.
